# The Influence of Motion on the Delivery Accuracy When Comparing Actively Scanned Carbon Ions versus Protons at a Synchrotron-Based Radiotherapy Facility

**DOI:** 10.3390/cancers14071788

**Published:** 2022-03-31

**Authors:** Franciska Lebbink, Markus Stock, Dietmar Georg, Barbara Knäusl

**Affiliations:** 1 MedAustron Ion Therapy Centre, Medical Physics, 2700 Wiener Neustadt, Austria; franciska.lebbink@medaustron.at (F.L.); markus.stock@medaustron.at (M.S.); 2 Department of Radiation Oncology, Medical University of Vienna, 1090 Vienna, Austria; dietmar.georg@akhwien.at

**Keywords:** 4D, particle therapy, breathing motion, motion mitigation

## Abstract

**Simple Summary:**

The interplay of breathing and beam motion reduces the efficacy of particle irradiation in moving tumours. The effect of motion on protons and carbon ion treatments was investigated dosimetrically and the results were benchmarked against each other by employing an anthropomorphic thorax phantom that was able to simulate tumour, rib, and lung motion. The critical question was whether target coverage and organ-at-risk sparing could be maintained when the application of simple motion mitigation was addressed. Special focus was put on unique synchrotron characteristics, such as pulsed beam delivery and beam intensity variations. It could be demonstrated that the effect of motion was greater for carbon ions than for protons. These findings demonstrated the need for applying motion mitigation techniques depending on the motion amplitude, particle type, and treatment prescription considering complex time correlations.

**Abstract:**

Motion amplitudes, in need of mitigation for moving targets irradiated with pulsed carbon ions and protons, were identified to guide the decision on treatment and motion mitigation strategy. Measurements with PinPoint ionisation chambers positioned in an anthropomorphic breathing phantom were acquired to investigate different tumour motion scenarios, including rib and lung movements. The effect of beam delivery dynamics and spot characteristics was considered. The dose in the tumour centre was deteriorated up to 10% for carbon ions but only up to 5% for protons. Dose deviations in the penumbra increased by a factor of two when comparing carbon ions to protons, ranging from 2 to 30% for an increasing motion amplitude that was strongly dependent on the beam intensity. Layer rescanning was able to diminish the dose distortion caused by tumour motion, but an increase in spot size could reduce it even further to 5% within the target and 10% at the penumbra. An increased need for motion mitigation of carbon ions compared to protons was identified to assure target coverage and sparing of adjacent organs at risk in the penumbra region and outside the target. For the clinical implementation of moving target treatments at a synchrotron-based particle facility complex, time dependencies needed to be considered.

## 1. Introduction

The interplay effect [1] describes the accuracy degradation of the dose delivery for scanned pencil beams when irradiating moving targets. Additional range deviations are caused by organ motion due to changing tissue densities in the beam path. These effects cause under- and overdosage in the target region and/or unwanted irradiation of nearby organs at risk (OARs) [1]. Due to basic physical properties, such as lateral scattering, the fragmentation tail, and spot sizes, carbon ion spot distributions are especially affected. Classical motion mitigation techniques, such as breath-hold, gating, and tracking can potentially compensate for these effects [1]. The most applied method in scanning facilities is planning on a breathing-adapted target volume with margins, potentially supplemented by gating at a specific breathing phase or rescanning [2]. Prior clinical implementation is essential for performing extensive and centre-specific tests of the required components, as well as end-to-end tests [3].

Passive scattering [4] and active scanning [5] with carbon ions has been used to treat a large variety of tumours in Japan during the last 20 years, and carbon-ion therapy centres in Japan work at the frontier of 4D-dose delivery [6,7,8,9]. Applying phase-controlled rescanning or respiratory gating with active scanning poses a high level of complexity [10] because the treatment of moving tumours is not only sensitive to breathing, but also to the time structure of the accelerator.

Experimental validation of dose delivery to moving targets, as well as extensive retrospective analysis, are required to fully exploit the accuracy of ion beam therapy, especially at synchrotron-based active scanning facilities.

Besides classical motion mitigation techniques, changes of the beam delivery dynamics and spot characteristics might reduce the interplay effect and increase the planned robustness towards intrafractional anatomical changes [11]. As beam time and personnel resources are scarce and costly, in silico investigations could be the first choice to show the clinical necessity of motion mitigation. For proton therapy, 4D dose tracking (4DDT) was established and validated experimentally [12,13,14], while for carbon ions factors such as the particle spectrum, linear energy transfer (LET), and non-linearity with doses pose large challenges for biologically-weighted 4DDT [15,16].

Hence, measurements should be the ground truth for the implementation of motion mitigation techniques for carbon ions, which could also serve for validation of 4DDT at a later stage. For a more detailed understanding of the dosimetric effects during dose delivery to a moving system, time-resolved dosimetry with a high sampling rate using point measurements or employing ionization chamber arrays is the ideal choice [12,17,18,19].

This study tackled, for the first time, open questions in motion mitigation with actively scanned carbon ions employing time-resolved dosimetry in an anthropomorphic breathing thorax phantom. Carbon measurements were compared for different motion scenarios and proton measurements. In addition, the influence of rescanning and spot characteristics on the dependence of the accelerator delivery dynamic was explored.

## 2. Materials and Methods

### 2.1. Treatment Facility

Carbon ion and proton measurements were performed in the horizontal beam line at MedAustron Ion Therapy centre (MedAustron), a synchrotron-based active scanning facility. A spill length of 4 s (carbon ions) or 5 s (protons) and a constant energy switching time of 2 s characterised the pulsed beam delivery. Nominal maximal dose rates of 1.2 × 10^8^ carbon ions per s and 1.8 × 10^9^ protons per s were possible. To assure the highly precise delivery of a treatment plan, the dose delivery system (DDS) monitored the spot positions and intensities, which required a minimal spot-on time. This could only be facilitated by beam transmission degradation (deg) and a minimum number of particles per spot. The degradation, which is a facility-specific issue in beam generation, was achieved by a perforated plate that blocked a certain fraction of particles before entering the main accelerator ring and limited the upper amount of particles in a spill. To reduce the risk of overdosage in some failure scenarios of the accelerator a deg of up to 20% (deg20) was clinically used.

The total treatment time was 3–4 min for a tumour volume of 100 cm^3^, which was comparable for protons and carbon ions. The irradiation room was equipped with an imaging ring (ImagingRing™, medPhoton GmbH, Salzburg, Austria) [20], a tracking camera, and a patient couch, which was controlled by a robotic arm (Exacure, BEC GmbH, Pfullingen, Germany) [21].

### 2.2. Phantom, Motion Amplitudes, Dosimetry, and Experimental Setup

**Figure 1:**
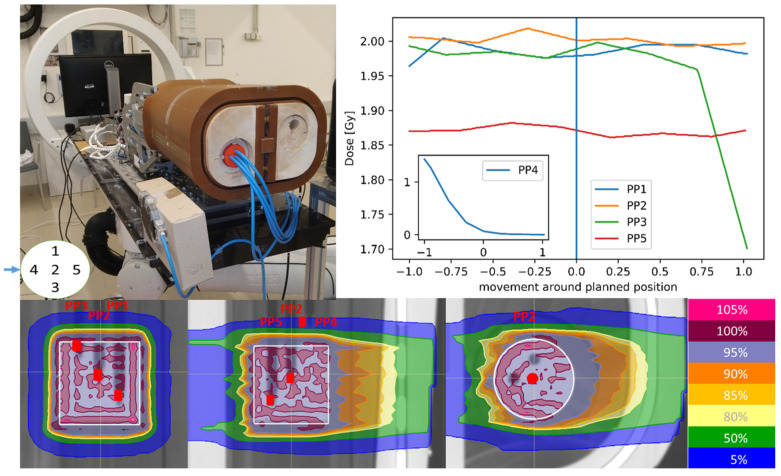
Setup with ARDOS (**left**) and line dose from the carbon ion plan through the PinPoint chambers around the planned position (**right**) with dose profiles from the carbon ion plan (**below**). The red dots show the location of the different PinPoint chambers.

The in-house-developed Advanced Radiation DOSimetry phantom (ARDOS) (Figure 1) [12,22,23] was utilised. The phantom represents the human thorax anatomy, consisting of solid water for mimicking soft tissue, cortical bone substitute for the ribs, and balsa wood for lung tissues. With four independent motion axes for the tumour (translation and rotation), realistic rib and lung expansion breathing patterns were simulated with submillimeter accuracy and reproducibility. The trigger signal from the accelerator started the ARDOS movements, which were logged in files generated by digital linear position string encoders attached to the motion axes of the phantom.

Patient-like motion amplitudes, as observed for clinically treated lung patients, were implemented for the different phantom axes, varying in motion amplitude and in combinations of tumour movement with or without rib and/or lung expansion. The following motion scenarios were investigated with a breathing period of 5 s for protons and carbon ions and beam transmission degradation of 20 (deg20): tumour motion of 0.6, 1, 1.5, and 2 cm perpendicular to the beam in combination with the rib-opposed direction (3 mm) and/or lung expansion (2 mm).

For all measurements, the motion scenarios started on the respective full exhale phase, unless specified otherwise. The effect of starting in the full inhale phase and a slower delivery speed (beam transmission degradation 10 (deg10)) were investigated further for the 2 cm tumour movement for both particle species.

Carbon ion measurements were additionally extended with rescanning and range-shifter treatment plans for tumour motion amplitudes of 0.6 cm and 2 cm without any rib/lung motion (more details described in Table 1).

The ARDOS phantom was positioned with a fixed nozzle distance of 66.1 cm in a way that the horizontally oriented beam was coming from the side, perpendicular to the superior–inferior motion direction, as depicted in Figure 1. The correction of the patient couch position (6 degrees of freedom (DoF)) was calculated based on two orthogonal X-ray images in combination with digitally reconstructed radiographs (DRRs) of the planning computed tomography (CT) [20], which assured the correct ARDOS positioning for each measurement day. The ARDOS phantom was connected to the accelerator by an optical fibre to synchronize the start of the phantom with the extraction of the beam.

The dose was measured inside the tumour and lung material of the ARDOS with five 0.03 cm^3^ PinPoint (PP) ionisation chambers (type TM31015, PTW, Freiburg, Germany) at different positions, denoted PP1 to PP5, connected to a MULTIDOS multichannel electrometer (SN1584, PTW, Freiburg, Germany) as described in [12]. The measurement positions were assigned to different regions describing the location relative to the tumour as follows, which were later considered in the evaluation:

- Inside the tumour, PP1, PP2 and PP5;
- In the penumbra region of the tumour, PP3;
- Outside the tumour in the lung tissue, PP4.

Time-resolved dosimetry logfiles were recorded in 0.5 s intervals. Measurements were performed on seven different days (2–3 repetitions per day) while the number of measurement repetitions differed for the different motion scenarios and particle types, which are listed in detail in Table 2. The delivery time, extracted from accelerator log files, depended on the dose rate, as shown in Table 1. The beam delivery of the synchrotron, the ARDOS movements, and the time-resolved measurements were synchronised to determine the effect of the motion and the accelerator time structure on the treatment outcome.

### 2.3. Imaging and Treatment Planning

CT images of the ARDOS were obtained by a Philips CT scanner (Brilliance CT Big Bore Oncology, Philips, Best, The Netherlands) and the clinical acquisition protocol for the abdomen with a voltage of 120 kV, an exposure of 300 mA s, and a slice thickness of 3 mm. A CT scan of the static phantom with a tumour position corresponding to the mid ventilation breathing phase was employed as a reference CT phase, later called the planning CT, which was used for spot distribution and weight optimisation.

Carbon ion and proton treatment plans were created on the planning CT in RayStation 7.99 (RaySearch Laboratories AB, Stockholm, Sweden) with a 2 mm^3^ calculation grid aiming for a homogeneous coverage of the median dose to the target with a single horizontal beam from the right (Figure 1). The target was delineated as a cylinder within the lung tissue with a diameter of 5 cm and a height of 5 cm corresponding to the size of the solid water tumour replica. Pencil-beam (PB) dose calculations were employed for the carbon ions (PB v3.0) and Monte Carlo (MC) dose calculations for the protons (MC v4.3), based on the Hounsfield units (HU) to stopping power calibration curve commissioned for the clinical CT protocol for abdominal imaging [24]. The mean biologically weighted prescribed dose was 5 Gy for the carbon ions and 2 Gy for the protons. All details of the spread-out Bragg peak (SOBP) plans are given in Table 1.

The standard carbon ion and proton treatment plans were created with deg20. Additional plans were created with deg10 allowing for a lower minimum number of particles per spot, a 3 cm range shifter in the beam path, and layer-rescanning (maximal number of particles of 50 × 10^6^ NP per layer) (shown in Table 1). The different minimum number of particles required per spot resulted in mixed degrader settings (deg10 and deg20) for the rescanned plans.

The use of a range shifter led to bigger spots and decreased the dose conformality, although it could improve the dose homogeneity in moving tumours depending on the material and air gap [10,25]. The given phantom characteristics and the setup with a fixed nozzle distance achieving a homogeneous dose to the target with the range shifter required more energy layers and spots compared to the deg20 carbon treatment plan.

### 2.4. Evaluation Tools and Statistics

Absolute dose values and reported dose deviations for all motion scenarios were compared to the respective static measurements. An unequal variance *t*-test, Welch’s *t*-test, was performed (significance level *p* < 0.05) to compare the different motion scenarios to the static scenario and to each other, as well as to compare proton and carbon-ion measurements.

## 3. Results

Section 3.1 describes the effect of different motion amplitudes of all phantom components under reproducible free breathing conditions without any motion mitigation techniques for protons and carbon ions. For carbon ions the benefit of simple modulations in the beam characteristics and delivery dynamics, such as layer-rescanning and enlarged spot sizes, are analysed in Section 3.2 for 0.6 cm and 2 cm tumour motions only.

### 3.1. Effect of Motion

**Figure 2:**
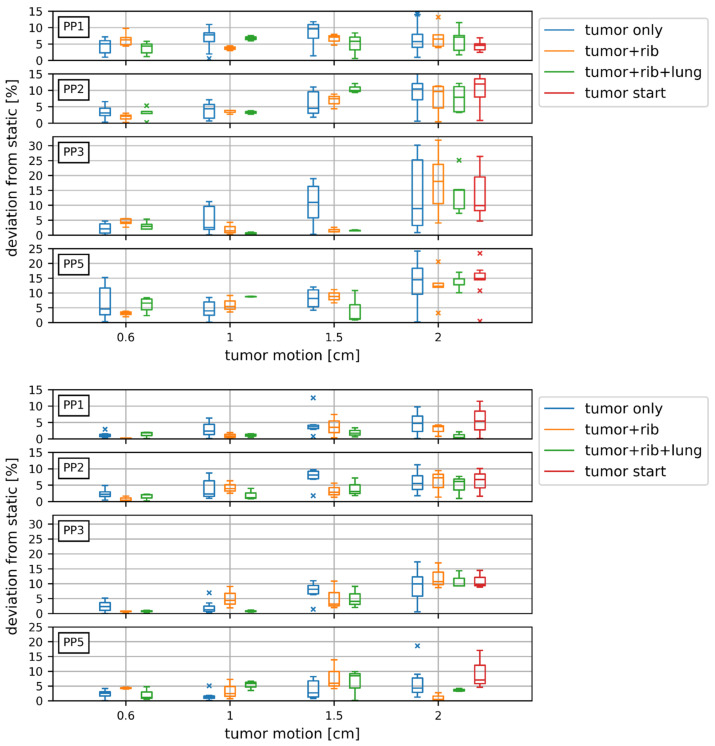
Boxplots containing the deviation from the static dose for different motion scenarios (tumour motion of 0.6, 1, 1.5, and 2 cm with or without a combination of rib and lung motion) for carbon ion (**upper**) and proton (**lower**) irradiations. The red boxplot represents different breathing starting phases.

For carbon ions, the motion of the tumour only resulted in a significant dose disturbance compared to the static scenario for PP2, PP3, and PP4 for 7 out of 12 motion amplitudes, despite the large dose rate variations (shown in Figure 2 and Table 2). With an increasing tumour motion amplitude from 0.6 cm to 2 cm the dose deviation increased inside the target from 4.5% ± 2.1% (*p* < 0.05) to 6.5% ± 4.3% (*p* < 0.05) for PP1 and from 3.5% ± 1.7% (*p* < 0.05) to 9.8% ± 4.6% (*p* < 0.05) for PP2. At the distal end of the target the significant dose deviations exceeded 10% (PP5). For the penumbra region (PP3) the dose deviation increased from 2.3% ± 1.6% (*p* < 0.05) to 13.6% ± 10.8% (*p* < 0.05) due to the steep dose fall off as depicted in Figure 1. PP4 in the distal target region received less than 0.05 ± 0.02 Gy in the static scenario but increased up to 0.2 ± 0.1 Gy (*p* < 0.05) with 2 cm tumour motion.

A comparison of the different tumour motion amplitudes showed that for PP2, PP3, PP4, and PP5 a tumour motion of 2 cm had a significantly (*p* < 0.05) higher impact on the dose values than a tumour motion of 0.6 cm.

Adding the rib as well as rib + lung motion, or changing the breathing starting position, did not result in any significant difference considering the dose deviation relative to the static measurements, as depicted in detail in Figure 2, e.g., the mean values of the dose deviation for PP2 in the centre of the target with 2 cm motion were 9.8% ± 4.6% for tumour motion alone, 7.4% ± 4.3% adding the rib motion, and 7.6% ± 3.7% adding rib + lung motion. Changing the starting position resulted in a dose deviation of 10.3% ± 4.4%.

For proton irradiations, the dose on the central axis of the tumour perpendicular to the beam (PP1, PP2) did not vary significantly for all motion scenarios with a tumour motion of 0.6 cm. For the distal target region (PP5) no deviations larger than 5% were observed for these small movement scenarios compared to static (tumour only and rib *p* < 0.01 and rib + lung *p* > 0.05). For PP3 the 0.6 cm tumour motion caused a dose distortion within 5% compared to the static scenario, as shown in Figure 2.

For all 2 cm tumour movement scenarios (including the scenarios with rib and rib + lung), PP1, PP2, and PP5 inside the tumour deviated significantly by up to 6% from the static plan, while this increased to 12% for the penumbra region (PP3). For PP4 a 2 cm tumour movement scenario induced a dose deviation of up to 16% (Table 2).

#### 3.1.1. Comparison of Protons and Carbon Ions

**Figure 3:**
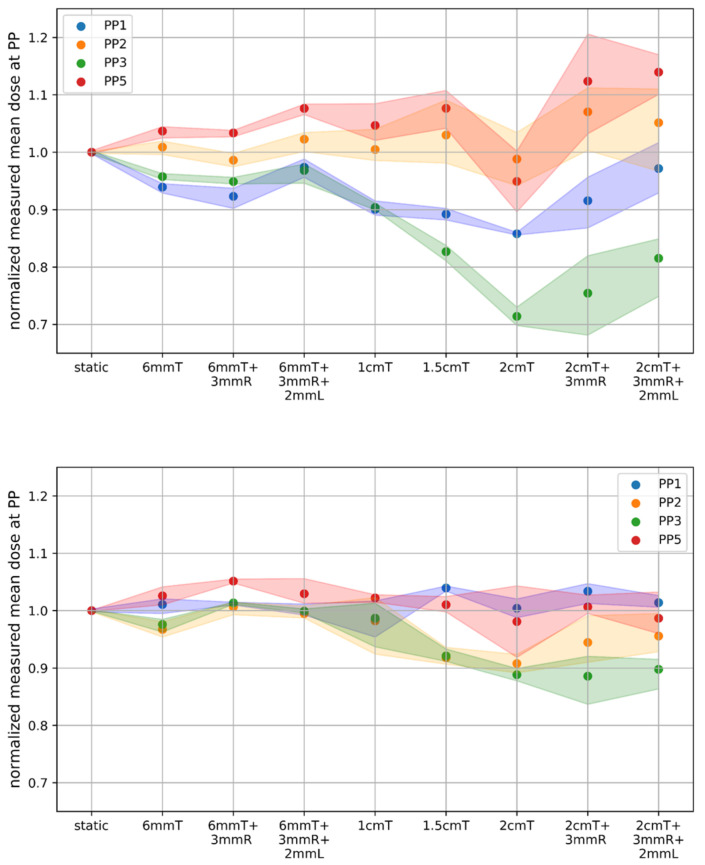
Normalized carbon ion (**upper**) and proton (**lower**) dose at [PP1-3,5] for the different motion scenarios during one measurement day. The different colours represent the different PinPoint chambers. The shaded area around the points shows the maximum and minimum within the scenarios for the repeated measurements. T = tumour movement, R = rib movement, and L = lung expansion.

The impact of motion was higher for carbon ions than for protons. The maximum deviation from the respective static measurement was smaller for all motion scenarios for protons compared to carbon ions, as listed in Table 2, e.g., 30% (*p* < 0.05) for scenarios with 2 cm tumour movement for carbon ions compared to 17% (*p* < 0.05) for protons. Ad- ditionally, the standard deviation of the measurement repetitions decreased for protons compared to carbon ions, even the variation in the delivery time was bigger (Table 1). For illustrating the effect of the different motion amplitudes for protons and carbon ions all motion scenarios from measurement day 1 were plotted in Figure 3.

#### 3.1.2. Time-Resolved Analysis

Variations in dose for the different measurement repetitions could be related to dose rate fluctuations, while the influence was weaker for smaller motion amplitudes. Including all eight measurement days, the delivery time ranged from 165 s to 210 s for the deg20 carbon ion plan (Table 1) with a maximum variation of 2 s for a single day. Naturally this influenced the amount of the 5 s breathing cycles per measurement, which varied between 33 and 41.5.

**Figure 4:**
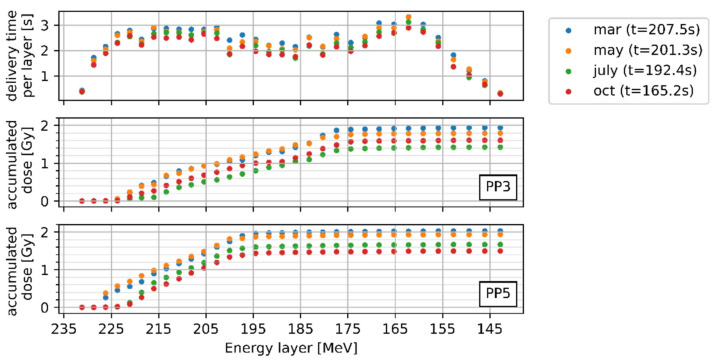
Delivery time per energy layer and dose for PP3 and PP5 for the different energy layers on four measurement days; static dose values were: PP3 = 1.97 ± 0.01 Gy and PP5 = 1.85 ± 0.01 Gy; t gives the total delivery time of each measurement day varying between 165.2 s to 207.5 s.

The effect of this fluctuation on the dosimetric outcome is depicted for PP3 and PP5 in Figure 4, where four selected measurements with a 2 cm tumour motion (at four different time points) have been compared. Analysing the effect of the dose rate in detail revealed a complex time correlation between the breathing cycle, dose deposition per energy layer, and also the total delivery time. For example, in the third energy layer (E = 226.2 MeV) the accumulated dose for PP5 was up to 0.4 Gy for the longer delivery times, while for faster delivery times it was below 0.05 Gy. The total accumulated dose varied between 1.42 Gy to 2.09 Gy for PP5 and between 1.43 Gy to 1.94 Gy for PP3. The same behaviour was observed for PP1 and PP2 with a smaller dose difference (up to 0.25 Gy) between the different measurements. Changing the starting position of the tumour motion did reveal comparable dose differences (0.61 Gy for PP5 and 0.38 Gy for PP3) considering the respective time points.

### 3.2. Influence of Beam Delivery Dynamics and Spot Characteristics

**Figure 5:**
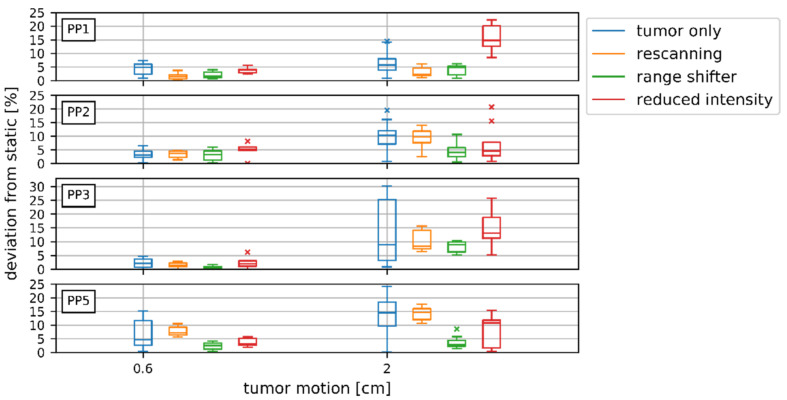
Boxplots containing the deviation from the static dose for different motion scenarios (tumour motion of 0.6 and 2 cm) for carbon ion irradiations with different motion mitigation techniques.

A reduction in the beam transmission intensity from deg20 to deg10 altered the dosimetric outcome, but no significant difference was observed for protons considering all PP chambers. For carbon ions deg10 and deg20 resulted in comparable dose deterioration compared to the static scenario. The doubling of the delivery time resulted in a significant increase in the dose deterioration for 2 cm tumour motion from 6.5% ± 4.3% to 15.8% ± 4.8% only for PP1, depicted in Figure 5.

The rescanned treatment plan consisted of a mixture of beam transmission intensities. Hence, the effect of layer rescanning was compared to deg20, as well as to deg10, and revealed different results for the investigated scenarios. Inside the tumour the dose deviation compared to the static scenario was reduced by more than 50% when rescanning was applied, compared to deg20 (e.g., for PP1 from 6.5% ± 4.3% to 3.1% ± 1.8% (*p* < 0.05)) for a 2 cm tumour motion amplitude. The comparison with deg10 showed an even larger decrease in the dose distortion from 15.8% ± 4.8% to 3.1% ± 1.8% (*p* < 0.05) for PP1 and from 14.3% ± 2.3% to 8.6% ± 5.5% (*p* < 0.05) for PP5 for a 2 cm tumour motion amplitude. This observation posed the assumption that the beam intensity settings had a bigger influence than the rescanning itself.

The use of different beam characteristics employing a bigger spot size changed the initial dose distribution, which, essentially, resulted in an increased dose outside the tumour for PP4 from 0.04 ± 0.02 Gy to 0.16 ± 0.08 Gy for the static scenario (*p* < 0.05). For the range shifter plan the 0.6 cm tumour movement resulted in a deviation of the dose up to 3% compared to the static scenario. For a motion amplitude of 2 cm the deviation was 3.9% ± 1.9% for PP1, 4.5% ± 3.1% for PP2, 8.2% ± 2.0% for PP3, and 3.7% ± 2.2% for PP5 compared to the static range shifter plan, which was a significant decrease in the dose distortion (*p* < 0.05) compared to the deg20 plan with the same motion. In general, increasing the spot size reduced the dose distortion and the results were more comparable to the static scenario.

Rescanning and increasing the spot sizes led to less variation within all the scenarios, which confirmed the expectation of higher plan robustness with respect to motion.

## 4. Discussion

In this study, the combination of an anthropomorphic breathing phantom and time-resolved dosimetry was employed to determine the effect of beam and organ motion on the dose delivery accuracy of proton and carbon ions. In contrary to a study previously published by our group [12], ribs were added to the ARDOS mimicking a more anthropomorphic situation resulting in slight dosimetric differences, especially with respect to significance levels. Not only the presence of the ribs but also their motion was investigated in detail.

The time-resolved dosimetry framework gave insight into the dose delivery dynamics related to beam intensity variations and target motion and served as the basis for the analysis of the variation in accumulated doses. Intensity variations observed during measurements, e.g., from 1 × 10^8^ NP/s to 5 × 10^8^ NP/s for protons, also occurred clinically where the NP/s varied by a factor of two for protons and three for carbon ions during the same period of one year. The beam extraction duration changed between July and October from 32 s to 9 s, essentially influencing the accelerator-triggered synchronisation between phantom movement and spot delivery. This study showed the importance of investigating the impact of such accelerator performance upgrades, which are likely to happen in synchrotron-based facilities.

The fluctuating dose rate and the extraction duration caused different phantom positions for the irradiated energy layers, e.g., for the sixth layer in Figure 4, the target moved between −1 cm to 0.2 cm in May, while it moved from −0.5 cm to 1 cm in October. Once gated beam delivery is available, the difference in extraction duration will be overcome, paving the way towards a clinical end-to-end test.

The temporal resolution of the analysis was mainly limited by the PP chamber read- out frequency of 0.5 s (average delivery time per energy layer: 2.0 ± 0.7 s). Accelerator log files documented the irradiation progress every 0.05 s and in addition the starting time of every spot. The retrospective synchronisation of the accelerator log file information with the PP chamber readout was based on the 0.001 Gy threshold of the central PP chamber (PP2). The highly accurate synchronisation become especially important once the presented measurements serve for the validation of the carbon ion 4DDT.

The spatial resolution and the number of PP chambers potentially reduced the overall sensitivity of the analysis. The number of PP chambers within the small tumour volume could not be increased as shadowing of the chambers needed to be carefully avoided. PP chamber measurements in the static phantom were reproducible and acquired within 0.5% for all measurements in a period of one year reflecting the highly accurate and reproducible X-ray-guided setup and proving the sufficient accuracy with respect to dose heterogeneity in the static scenario. The partial irradiation of the PP chambers, especially in the penumbra region, might have influenced the dosimetric accuracy of the measurements during motion. With a sensitive volume of 0.03 cm^3^ and a motion speed of 0.8 cm/s, PP3 in the penumbra region of the target (Figure 1) was partially moved out of the irradiation field in 12.5% of the delivery time. This could have potentially led to a lateral electronic disequilibrium affecting the conversion of signal into dose-to-water in the detector [26]. The directional response of the PP chambers, which was smaller than 0.5% for rotation around the chamber axis, was carefully prevented by fixing the chambers with a screw in the insert.

Most studies investigating the effect of motion experimentally are based on 2D measurements with a chamber array placed on top of a moving platform [16,27,28,29]. The study presented here combines the motion of multiple PP chambers at different depths with a realistic moving anatomy, expanding the currently available dosimetric investigations to four dimensions (space and time). The phantom parts were based on geometric shapes in order to guarantee highly accurate and reproducible measurements and to represent a big patient cohort rather than a specific patient collective. Steinsberger et al. [16] investigated the same breathing period (5 s) and amplitudes (1 cm and 2 cm lateral detector movement) for carbon ions using a 2D ionization chamber array. The gamma pass rate (2%/2 mm) of the 2 cm motion amplitude was 90%, which was comparable to our measurements since the average dose disturbation was up to 10% for 2 cm tumour motion.

Lis et al. [29] performed squared single energy carbon field measurements with a moving platform in combination with homogeneous tissue-equivalent materials to investigate the effect of gating. The gamma pass rate with a 3%/3 mm criterion could be improved from 44% to 96% for 2 cm motion and a 4 s breathing period when gating was applied. Extending these moving platform measurements by a wedge and a heterogeneous slab phantom in combination with ionisation chambers [30] showed a dose distortion of up to 30%. Such a strong effect of motion was not observed in our study delivering SOBP, where the maximal distortion of 30% was only observed for outliers. As shown by the time-resolved analysis, the clinically relevant effect of motion on the dose at different positions within and outside the target was strongly affected by the particles’ ranges and might not be reflected by single energy layer measurements.

In the presented study the proton measurements for a motion amplitude of 0.6 cm to 1.0 cm showed a dose distortion within 5% for the central PP chambers. These results confirmed the findings of anthropomorphic phantom measurements [31], where five radiochromatic films (type EBT3) were inserted within a spherical tumour of 6 cm. For 1 cm motion, the gamma pass rate analysis (4%/4 mm) for the central planes was >90%. The higher deviations could be explained by the larger motion amplitude uncertainty (0.5 cm) and a setup uncertainty of 0.3 cm, as reported by Perrin et al. [31]. Pfeiler et al. [27] observed a maximum deviation of 4% shooting protons through an inhomogenous tissue equivalent plug phantom positioned on a MatriXX (IBA Dosimetry, Schwarzenbruck, Germany) moving with an amplitude of 0.5 cm (CIRS dynamic phantom, CIRS, Norfolk, VA, USA).

The effect of rescanning for actively scanned protons [32] and carbon ions [16] was previously investigated employing 4D dose calculations resulting in an increase in D_95%_ by more than 5% for different motion amplitudes, rescanning schemes, and number of repetitions. This reduction in dose distortion could also be observed in our study for 2 cm motion, where the carbon ion dose distortion decreased by 3.4% when employing layer-rescanning. As was already shown for protons, the relation between the number of rescans and degree of dose homogeneity was non-linear due to the interference between the motion period and the period of rescanning [33]. For carbon ions the potential of rescanning might be mainly determined by the delivery dynamics (details in Section 3.2), as the treatment time needs to be kept as short as possible.

The potential of bigger spot sizes has been reported for protons in the literature, where the target homogeneity for moving targets could be increased by a factor of 2–3 for enlarged spot sizes [34,35]. This study showed that the use of a range shifter, which increased the spot size, also improved the robustness of the treatment delivery towards motion for carbon ions, although the initial target homogeneity was decreased. More specifically the deviation within the target area could be reduced by a factor of two by investigating 2 cm tumour motion.

The broadening could be reduced by moving the patient closer to the range shifter, preventing degradation of the treatment plan quality [36], but the complexity of our setup did not allow for a varying air gap.

The motion of heterogeneous components in the beam path was especially investigated in this study. While the presence of ribs was shown to be essential, their motion (alone or in combination with lung expansion) did not show a significant difference compared to tumour motion alone. The rib density of 1.82 g/cm^3^ [22] in the ARDOS phantom was higher compared to real anatomical ribs, while the lung density was comparable to clinical data. Anyhow, real lung tissue might lead to a ripple filter effect, further mitigating the effect on the dose distribution [37].

A limitation of this study was the use of a horizontal beamline only. Employing multiple beams, applying a table or beam rotation, and gating might mitigate the effect of motion but might also increase the heterogeneity of the dose distribution of a single carbon beam. Further investigations based on clinical treatment concepts will be necessary to investigate this effect in more detail. Once 4DDT is established for carbon ions, the acquired measurements will be employed for validation of the 4DDT framework in a similar approach as presented by our group recently [12].

## 5. Conclusions

For carbon ions, a small tumour motion of 0.6 cm disturbed the static dose distribution significantly, which increased for higher motion amplitudes, especially the dose in the penumbra region and outside the target, which was heavily modulated by movements. While the motion of the ribs and lungs did not additionally deteriorate the dose, a high sensitivity to dose rate fluctuations was observed. The measurements confirmed the need for motion mitigation for carbon ions, while both layer-rescanning and an increase in spot size were able to reduce the dose distortion.

For protons the necessity for motion mitigation could not be proven for tumour amplitudes smaller than 1 cm but they need to be considered for a motion amplitude of 2 cm.

Before clinical implementation of moving target treatments at a synchrotron-based particle facility, especially with carbon ions, complex time correlations need to be considered and even gated beam delivery could be possible.

## Figures and Tables

**Table 1:** Delivery time and number of energy layers and spots, as well as energy range and number of particles per fraction for the different proton and carbon ion treatment plans. The delivery time during a measurement day varied by around 2 s, while the delivery time in the table shows the variation between the different measurement days.

	Proton		Carbon Ion			
Plan	deg20	deg10	deg20	deg10	Rescanning	Range Shifter
Beam transmission degradation	20%	10%	20%	10%	mixed	20%
Delivery time [s]	235–272	544–563	165–210	314–326	265–269	244–254
No. of energy layers	47		32		58	63
Beam meterset [10 × 10^6^ NP/fx]	59,898		2621			3157
Min. Energy	73.3 MeV		142.8 MeV			193.1 MeV
Max. Energy	124.7 MeV		231.2 MeV			272.5 MeV
No. of Spots	5772		11,477			16,346
Spot spacing [cm]	0.27–0.32		0.26–0.28			0.24–0.28

**Table 2:** Average dose values and their related standard deviation (SD) for the different PP locations for the carbon and proton measurements; additionally, the absolute maximal dose deviation is given, the asterisk (*) means statistically significant compared to the static measurement (*p* < 0.05), *n* is the total number of measurements, and ‘days’ is the number of measurement days.

Dose [Gy]							Max. Dose Deviation [Gy]			
Tumour Motions										
PP		Static	0.6 cm	1 cm	1.5 cm	2 cm	0.6 cm	1 cm	1.5 cm	2 cm
Carbon	1	1.96 ± 0.01	1.95 ± 0.10	1.95 ± 0.15	1.93 ± 0.18	1.95 ± 0.16	0.15	0.23	0.24	0.29
	2	1.95 ± 0.01	2.02 ± 0.04 *	2.00 ± 0.08	2.04 ± 0.10	2.11 ± 0.14 *	0.13	0.14	0.21	0.38
	3	1.97 ± 0.01	1.95 ± 0.04	1.90 ± 0.10	1.77 ± 0.14 *	1.70 ± 0.21 *	0.08	0.21	0.36	0.59
	4	0.05 ± 0.02	0.05 ± 0.02	0.08 ± 0.04 *	0.15 ± 0.11 *	0.18 ± 0.11 *	0.05	0.10	0.39	0.42
	5	1.85 ± 0.01	1.79 ± 0.14	1.93 ± 0.05 *	1.98 ± 0.10 *	1.76 ± 0.26	0.27	0.16	0.23	0.43
*n* (days)		18 (7)	15 (7)	10 (4)	8 (3)	15 (7)				
Proton	1	1.99 ± 0.01	1.99 ± 0.03	1.95 ± 0.06	1.99 ± 0.12	1.97 ± 0.11	0.05	0.12	0.25	0.18
	2	1.96 ± 0.01	1.93 ± 0.05	1.99 ± 0.09	1.87 ± 0.13	1.99 ± 0.13	0.10	0.16	0.19	0.22
	3	1.96 ± 0.01	1.91 ± 0.03 *	1.95 ± 0.06	1.87 ± 0.13	1.79 ± 0.10 *	0.10	0.13	0.21	0.34
	4	0.85 ± 0.17	0.80 ± 0.17	0.82 ± 0.16	0.82 ± 0.16	0.83 ± 0.16	0.26	0.22	0.22	0.24
	5	2.07 ± 0.01	2.11 ± 0.04	2.10 ± 0.04	2.07 ± 0.10	2.07 ± 0.16	0.08	0.10	0.17	0.39
*n* (days)		14 (5)	7 (3)	7 (3)	6 (2)	11 (5)				

## Data Availability

Not applicable.
